# Smart responsive organic microlasers with multiple emission states for high-security optical encryption

**DOI:** 10.1093/nsr/nwaa162

**Published:** 2020-07-09

**Authors:** Zhenhua Gao, Kang Wang, Yongli Yan, Jiannian Yao, Yong Sheng Zhao

**Affiliations:** Key Laboratory of photochemistry, Institute of Chemistry, Chinese Academy of Sciences, Beijing 100190, China; School of Materials Science & Engineering, Qilu University of Technology (Shandong Academy of Sciences), Jinan 250353, China; Key Laboratory of photochemistry, Institute of Chemistry, Chinese Academy of Sciences, Beijing 100190, China; Key Laboratory of photochemistry, Institute of Chemistry, Chinese Academy of Sciences, Beijing 100190, China; Key Laboratory of photochemistry, Institute of Chemistry, Chinese Academy of Sciences, Beijing 100190, China; University of Chinese Academy of Sciences, Beijing 100049, China; Key Laboratory of photochemistry, Institute of Chemistry, Chinese Academy of Sciences, Beijing 100190, China; University of Chinese Academy of Sciences, Beijing 100049, China

**Keywords:** organic laser, nanophotonics, excited-state process, responsiveness, optical encryption

## Abstract

Modern high-security cryptography and optical communication call for covert bit sequences with high coding capacity and efficient authentication. Stimuli-responsive lasing emissions with easily distinguishable readout are promising in the coding field as a novel cryptographic primitive, while the application is frequently restricted by the limited number of emission states. Here, we report a strategy of achieving multiple competitive lasing signals in responsive organic microspheres where a donor–acceptor pair was introduced. The competitive lasing from the donor and acceptor was reversibly switched by modulating the competition between the radiative rate of the donor and the rate of energy transfer, and the generated multiple lasing signals enabled a quaternary coding for recognizable cryptographic implementation. Data encryption and extraction were demonstrated using a 4 × 4 microlaser array, showing vast prospects in avoiding the disclosure of security information. The results offer a comprehensive understanding of excited-state dynamics in organic composite materials, which may play a major role in high-security optical recording and information encryption.

## INTRODUCTION

As photonic information technology rapidly develops, confidential tags with high density and security are urgently required and have attracted tremendous interest [1,2]. An effective approach to increasing the data security and storage density of such devices is the utilization of stimuli-responsive photoluminescent materials that can quickly switch among different states [3–11]. However, most of them can only provide broad-band photoluminescence (PL) from limited luminescent states, resulting in a relatively low security level [12]. In comparison with broad-band PL, stimulated emission with narrow linewidth for easily distinguishable readout [13] is promising in coding field as a novel cryptographic primitive [14–16]. Thus, developing smart responsive lasing materials, capable of rapidly switching among different emission states in response to external stimuli, is of great significance in the security area for their huge potential in preventing the disclosure of security information.

Organic materials with multiple upper-level structures and excited-state processes have been proven to possess tunable emission [17–21]. The lasing wavelength therefore can be tailored by controlling the population distribution on these upper levels. Nevertheless, owing to the limitation of the Franck–Condon principle, it has been a great challenge to broadly tailor the lasing wavelength [22,23]. The Förster resonance energy transfer (FRET) process involving multiple gain components provides an effective way to finely modulate multiple emission states [24]. The responsive FRET systems were commonly constructed by dynamically varying the distance between donors and acceptors through reversible manipulation of aggregation in a supramolecular film [25,26]. However, the methods of supramolecular control in these FRET systems usually suffer from laborious modulation procedures and/or large device footprints, which are unable to generate multiple lasing states in a microcavity, thus restricting their applications in high-density information storage and high-security optical encryption.

Herein, we propose a strategy to achieve multiple lasing states for high-security optical encryption by modulating the competition between the radiative rate of the donor and the rate of energy transfer in FRET microlasers. The competitive lasing signals from the donor–acceptor pair were reversibly switched, resulting in multiple distinguishable lasing states. The controlled lasing output enabled a quaternary coding and a proof-of-concept cryptographic implementation was demonstrated using a 4 × 4 microcavity array, which holds vast prospects in avoiding the disclosure of security information. The results not only offer a comprehensive understanding in excited-state dynamics in organic composite materials, but also open up a new way to construct flexible photonic components toward high-security optical recording.

## RESULTS AND DISCUSSION

**Figure 1.**
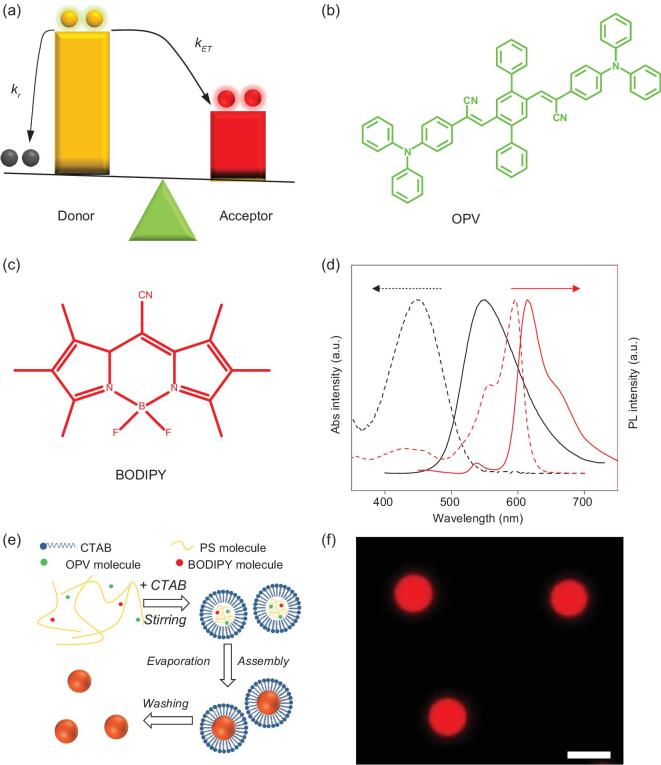
Design and preparation of the smart responsive organic microlasers. (a) Schematic diagram of the competition between the radiative decay of donor and energy transfer to the acceptor in a donor–acceptor pair. (b) Molecular structures of OPV donor molecules. (c) Molecular structures of BODIPY acceptor molecules. (d) Normalized absorption (dashed line) and fluorescence (solid line) spectra of OPV (black) and BODIPY (red) monomers dissolved in CH_2_Cl_2_. (e) Schematic diagram of the fabrication processes for OPV/BODIPY-doped microspheres. (f) PL image of microspheres with a doping molar fraction of 0.125 (BODIPY relative to OPV). Scale bar is 5 μm.

The smart responsive microlasers with multiple emission states were designed based on the FRET process, which has been utilized to finely modulate excitonic emission [27]. Figure 1a shows the scheme of the excited-state processes in organic composites where a donor–acceptor pair is involved. Upon absorbing a pump photon, the donor molecules undergo a transition from the ground state to high vibrionic levels of the first singlet excited state. Then it vibrationally cools fast to the bottom of the first singlet excited state. There are two major pathways that play a role in the following deexcitation of the singlet state: one is radiative decay to the ground state at the rate of *K_r_* and the other is energy transfer to the acceptor molecules non-radiatively at the rate of *K_ET_*. The radiative rate *K_r_* is highly dependent on the population of the first singlet excited state of the donor, which could be effectively controlled by the pump fluence, while the *K_ET_* remains approximately unchanged in a certain system. Thus, the competition between *K_r_* and *K_ET_* can be finely manipulated to enable different excitonic emissions [28,29]. Such multiple luminescent excited states could induce competitive lasing emissions because either the donor or the acceptor may lase when the corresponding population inversion is built up. These actively controlled multiple lasing emissions will significantly increase the storage density and data security, and thus are promising in avoiding information disclosure.

Here, 1,4-bis(α-cyano-4-diphenylaminostyryl)-2,5-diphenylbenzene (referred to as OPV, Fig. 1b and see Supplementary Figs 1 and 2) and 4,4-difluoro-8-cyano-1,2,3,5,6,7-hexamethyl-4-bora-3a,4a-diaza-s-indacene (referred to as BODIPY, Fig. 1c) were chosen as donor–acceptor model compounds because of their excellent lasing performances and large spectroscopic overlap between the absorption spectrum of the BODIPY and the fluorescence emission spectrum of the OPV that would facilitate energy transfer (Fig. 1d) [30,31]. Both the two compounds can be homogeneously doped into a flexible polymer matrix [32,33], which can readily assembly into microspherical resonant cavities to support laser emissions [34,35].

Spherical microstructures were prepared through an emulsion-solvent-evaporation method in a controllable way [36]. In a typical preparation (Fig. 1e), a well-mixed OPV–BODIPY/polystyrene(PS)/CH_2_Cl_2_ solution was added into cetyltrimethylammonium bromide (CTAB) aqueous solution. Under vigorous stirring, an oil-in-water emulsion was formed (see Supplementary Fig. 3). Driven by isotropic interfacial tension, PS molecules with low crystallinity prefer to aggregate into spherical structures after complete evaporation of CH_2_Cl_2_ solvent. The hydrophobic OPV–BODIPY molecules prefer to be embedded into the nonpolar PS spherical structures. The obtained microsphere composites have perfect circular boundaries and ultra-smooth surfaces (see Supplementary Fig. 4), which are favorable for whispering-gallery-mode (WGM) resonance (see Supplementary Fig. 5) and might trigger a low-threshold lasing. Under 420- to 480-nm light excitation corresponding to the OPV absorption band, the composite microspheres emit strong red fluorescence from the BODIPY (Fig. 1f). The red emissions are uniform over the whole microspheres (see confocal microscopy images shown in Supplementary Fig. 6), indicating that the acceptor molecules are well dispersed within the microspheres. The severe quenching of emissions from OPV molecules suggests an efficient energy transfer from the OPV donor to the BODIPY acceptor.

**Figure 2.**
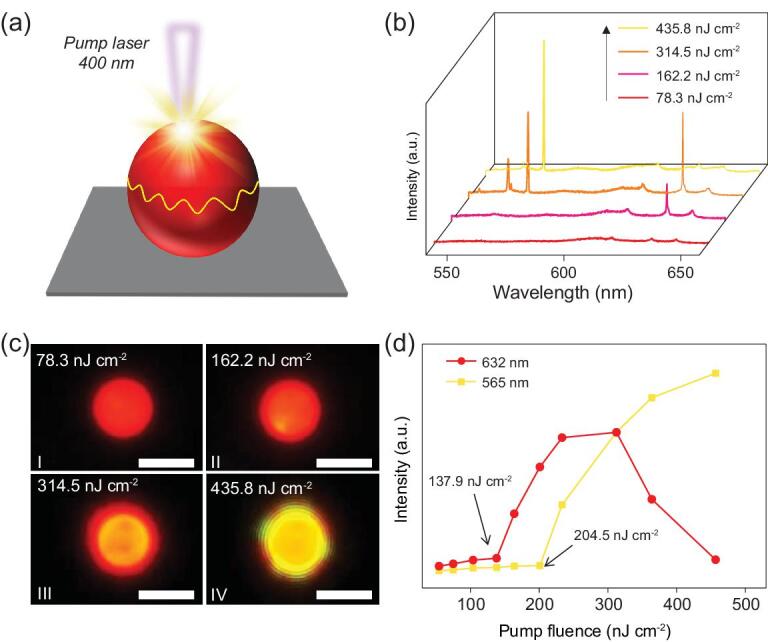
Competitive lasing performances from smart responsive microlasers. (a) Schematic illustration for the optical excitation of a single microsphere with a 400-nm pulsed laser. (b) PL spectra of an individual microsphere under different pump energies. (c) Corresponding real-color images of the microsphere excited with pulsed laser at different pump fluences (I–IV). Scale bars are 5 μm. (d) Power-dependent profiles of PL intensities around the mode peak 565 nm (yellow) and 632 nm (red), respectively.

The influence of FRET on the lasing process was investigated using a home-built far-field micro-photoluminescence (μ-PL) system (see Supplementary Fig. 7). A typical dye-doped microsphere was excited locally with a focused pulsed laser beam (Fig. 2a, 400 nm, 150 fs) and the PL signals under different pump fluences were collected. As shown in Fig. 2b, the PL spectrum at a low pump fluence (<78.3 nJ cm^−2^) is dominated by the broad spontaneous emission of the BODIPY. The absence of OPV emission (550–580 nm) should be attributed to the highly efficient energy transfer from the donor to the acceptor. With the increase in pump energy, several mode peaks located within the BODIPY emission band were selectively and strongly amplified, manifesting in the occurrence of lasing from the BODIPY (620–650 nm, 162.2 nJ cm^−2^). The FRET microlaser exhibits high photostability under laser operation, which may be attributed to the robust microcavity and efficient FRET process in the microspheres (see Supplementary Fig. 8). Under a high photoexcitation fluence (435.8 nJ cm^−2^), surprisingly, the lasing signal from the BODIPY disappeared. Instead, a series of new lasing peaks appeared in the emission band of the OPV molecules. Moreover, dual-wavelength lasing emission was achieved when a moderate pump fluence of ∼314.5 nJ cm^−2^ was employed. As shown in the corresponding real-color images (Fig. 2c), the color of the microsphere turns from red to orange and yellow gradually as the pump intensity increases, accompanied by the transition from the spontaneous emission of the acceptor to the distinct lasing signals of both donor and acceptor.

Figure 2d depicts the PL intensities at 565 and 632 nm as a function of the pump fluence, respectively. At first, the intensities of both emissions increase slowly with the increasing pump-pulse energy. Once the pump fluence rises above a certain value (137.9 and 204.5 nJ cm^−2^ for the acceptor and donor, respectively), superlinear increases in PL intensity are observed, which are assigned to the occurrence of stimulated emissions from the donor–acceptor pair. With further increase of pump fluence to >310 nJ cm^−2^, a gain saturation phenomenon is observed for the donor (yellow line) accompanied by the gradual decrease in the lasing intensity of the acceptor (red line), announcing that the lasing from the donor dominates the deexcitation processes of the donor–acceptor pair. At even higher pump fluences, the lasing signals from the acceptor totally disappear, while the lasing peaks from the donor continue to grow in intensity, revealing the suppression of the FRET process by the direct radiation of the donor.

**Figure 3.**
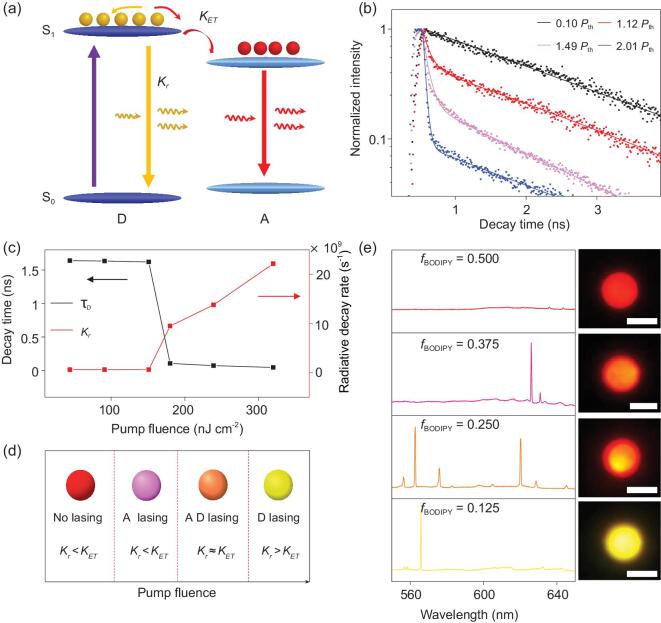
Mechanism of the competitive lasing emissions. (a) Schematic of excited-state processes in a donor–acceptor pair, showing the competition between radiative delay of the donor and energy transfer to the acceptor. (b) PL decay profiles of a typical OPV-doped microsphere monitored at 565 nm, illustrating the evolution from spontaneous emission to lasing emission with the increasing pump fluence. (c) Corresponding PL lifetimes (*τ*_D_) and decay rate (*K_r_*) of the OPV-doped microsphere monitored at 565 nm with the increasing pump fluence. (d) Schematic diagram of multiple emission states through modulating the competition between *K_r_* and *K_ET_*. (e) Lasing spectra of the microspheres with different doping molar fractions of BODIPY to OPV (*f*_BODIPY_) under a fixed pump fluence of 400 nJ cm^−2^. From top to bottom: *f*_BODIPY_ = 0.500, 0.375, 0.250 and 0.125, respectively. Insets: corresponding PL images of the microspheres. Scale bars are 5 μm.

The competitive lasing signals arise from the competition between stimulated emission from the donor and energy transfer from the donor to the acceptor, which is associated with the relative magnitude of *K_r_* and *K_ET_* (Fig. 3a). The *K_r_* is highly dependent on the population of the first singlet excited state of the donor, which can be effectively controlled through the excitation intensity. The dependence of *K_r_* on the pump fluence was explored through PL decay curves under varied pulse energy (Fig. 3b). When the pump fluence is smaller than the lasing threshold (*P*_th_, 136.5 nJ cm^−2^, see Supplementary Fig. 9), the PL emission follows a single-exponential decay with an average lifetime *τ*_D_ of 1.62 ns, corresponding to the spontaneous decay process (see Supplementary Table 1). Once the excitation fluence has increased above the lasing threshold, the PL signal turns to decaying biexponentially. Besides the initial slow component (∼1.6 ns), a new rapid component (<80 ps) emerges, which corresponds to the stimulated emission-induced excited-state-population depletion [37]. The decay process accelerated as the pump power increased, leading to the average decay time decreasing from ∼1.62 ns to 45 ps (Fig. 3c, black line).

Correspondingly, the *K_r_* (= 1/*τ*_D_) increased from 6.2 × 10^8^ to 2.2 × 10^10^ s^−1^ with the rising pump fluence (Fig. 3c, red line), while the *K_ET_* (∼1.3 × 10^9^ s^−1^, see Supplementary Fig. 10) was located within the scope of the *K_r_* changes. Therefore, the balance between *K_r_* and *K_ET_* synchronously changed with the pump fluence, which led to the generation of multiple lasing signals. The underlying mechanism behind the multiple lasing states is schemed in Fig. 3d. At a low pump fluence, there is no lasing emission as a result of insufficient gain. Because the *K_ET_* is larger than the *K_r_*, FRET dominates the deexcitation processes, leading to spontaneous emission from the acceptor. When the pump fluence exceeds the first lasing threshold (137.9 nJ cm^−2^), the majority of the excitation energy captured by donor transfers to the acceptor and lasing from the BODIPY occurs when population inversion is created. Further increasing of the pump fluence would induce simultaneous lasing emissions from the donor and acceptor as a result of the approaching of *K_r_* to *K_ET_*. At an even higher pump fluence, the *K_r_* outpaces the *K_ET_* and radiative decay from the donor begins to dominate the deexcitation processes, resulting in lasing from the donor when the corresponding population inversion is built up. Consequently, energy transfer to the acceptor is suppressed and lasing emission from the acceptor disappears due to the inefficient gain. Therefore, dynamic lasing action was well controlled through tailoring the balance between the radiative rate of the donor and the rate of energy transfer.

Besides *K_r_*, *K_ET_* is also very important in the manipulation of lasing signals. Considering that *K_ET_* is highly dependent on the donor-to-acceptor ratio [27], we investigated the *K_ET_*-dependent lasing in microspheres with varied molar ratios (*f*_BODIPY_, BODIPY to OPV), which are presented in Supplementary Fig. 11. It is shown that lasing emissions from both the donor and the acceptor were obtained from the microspheres with a low doping ratio of the BODIPY (*f*_BODIPY_ = 0.125 or 0.250), implying that the *K_ET_* is comparable with the *K_r_* in both cases. The *K_ET_* increases gradually with an increasing doping ratio. For microspheres with a higher doping ratio such as 0.375 or 0.500, high-density excitation only induces the BODIPY lasing as a result of a larger *K_ET_* than *K_r_*. Therefore, the microspheres with different doping ratios exhibit diverse emission states under a fixed pump fluence (Fig. 3e, from bottom to top panels), which should be ascribed to the distinct balance between individual *K_ET_* and *K_r_* values.

As mentioned above, multiple lasing states have been achieved in dye-doped microspheres through modulating either the pump intensity or doping the ratio of donor-to-acceptor molecules. Such multiple lasing states can potentially carry more information, providing a good platform for developing a quaternary coding resulting from the four possibilities of lasing states. The number of possible combinations in binary bits is 2*^n^*, while that in quaternary bits is 4*^n^* (*n* is the number of microlasers). Thus, the quaternary coding greatly enlarges the encoding capacity in comparison with the conventional binary counterpart. For example, an 8-bit quaternary code provides a theoretical capacity of 4^8^ (6.55 × 10^4^), which is 256 times higher than the magnitude of an 8-bit binary one (2.56 × 10^2^). More importantly, the quaternary coding could significantly enhance the security level of the cryptographic keys without increasing the physical size of the bit array (see Supplementary Fig. 12).

**Figure 4.**
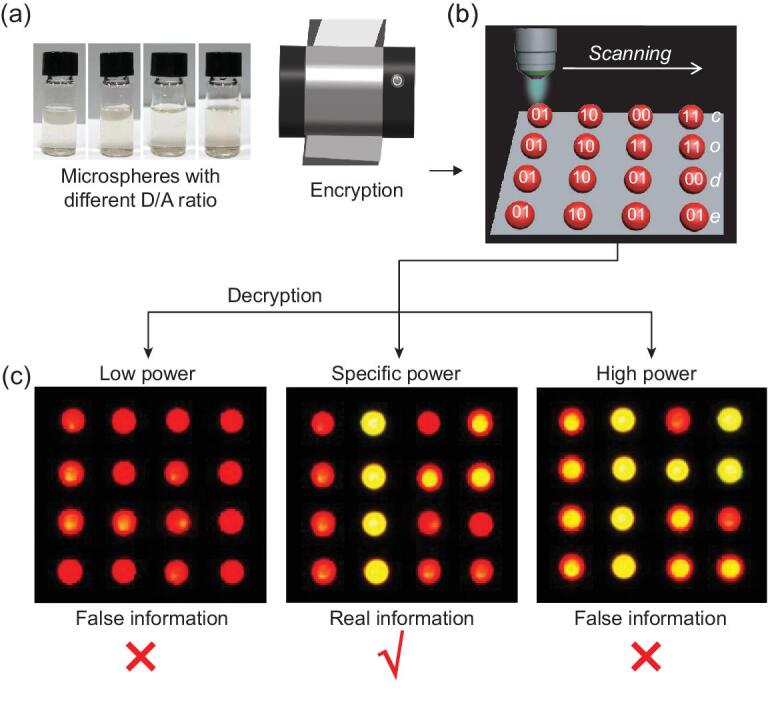
Proof-of-concept demonstration of the smart responsive microlasers for optical encryption. (a) Photographs of microspheres with different D/A ratios, which are used as encoding inks for information encryption through an inkjet printer. (b) Schematic illustration of authentication of photonic cryptographic primitives. The lasing signals can be effectively extracted by scanning the microlaser arrays. (c) Decryption of coded information through collecting the emissions from each microsphere. The real information could not be acquired unless a predesigned specific pump fluence (400 nJ cm^−2^) was adopted. Otherwise, the patterns could merely be translated into meaningless information.

The application of the smart responsive microlasers with multiple emission states in optical recording and identification is demonstrated in Fig. 4. As a proof-of-concept illustration, we set an encoding rule of ‘00’ for no lasing, ‘01’ for A lasing, ‘10’ for D lasing and ‘11’ for AD lasing, respectively, and meaningful information can be encoded into specific sequences of lasing signals. For example, the word ‘code’ consists of four letters and each letter is expressed by an eight-digit sequence [38]. Then, the word can be translated into a 4 × 4 microlaser array, where an individual microsphere in a specific position is determined according to the respective lasing signals under a predesigned ‘reading’ light. The generated microlaser array was fabricated using an inkjet printer with microspheres of different D/A ratios as inks (Fig. 4a).

The coded information is decrypted through collecting the emissions from each microsphere of the whole array using a reading beam (Fig. 4b). As mentioned above, the lasing signals are closely related to the pump fluence, thus the security information could not be identified whatsoever under a common daylight mode or an FL mode (under a UV lamp). To decrypt this information, a predesigned reading beam with specific fluence has to be used (Fig. 4c). Then, the lasing signals from individual microspheres were evaluated and the real information could be finally acquired. Otherwise, the patterns can merely be translated into meaningless information. This shows that multiple lasing signals effectively enhance the security of confidential information, which plays a significant role in avoiding information leakage.

## CONCLUSION

In conclusion, we have demonstrated a smart responsive microlaser with multiple emission states for high-security cryptographic implementation. The responsive microlasers, fabricated by incorporating a donor–acceptor pair into an identical microscale resonator, enable unique laser switching among multiple emission states of the donor–acceptor pair, which originates from the competition between the radiative decay of the donor and the energy transfer from the donor to the acceptor. On this basis, we realized a novel quaternary coding platform and a proof-of-concept demonstration of cryptographic application was exhibited with an inkjet-printed microlaser array. The results not only offer a comprehensive understanding of the function-oriented construction of organic composite materials, but also open up a new way for the fabrication of flexible photonic components that can be used for optical recording and information encryption.

## METHODS

The detailed preparation and characteristic methods of materials are available as Supplementary data at *NSR* online.

## Supplementary Material

nwaa162_Supplement_File
