# Supplementary material for: Smart responsive organic microlasers with multiple emission states for high-security optical encryption
Source: Natl Sci Rev. 2020 Jul 9;8(2):nwaa162. doi: 10.1093/nsr/nwaa162 (PMC8288339; doi:10.1093/nsr/nwaa162)
Supplement: nwaa162_Supplement_File [file nwaa162_supplement_file.pdf]

Supplementary data for

Smart Responsive Organic Microlasers with Multiple Emission States for High-Security Optical Encryption

Zhenhua Gao[1,2], Kang Wang[1], Yongli Yan[1]*, Jiannian Yao[1,3] and Yong Sheng Zhao[1,3]*

[1.] Key Laboratory of photochemistry, Institute of Chemistry, Chinese Academy of Sciences, Beijing 100190, China

[2.] School of Materials Science & Engineering, Qilu University of Technology (Shandong Academy of Sciences), Jinan 250353, Shandong Province, China

[3] University of Chinese Academy of Sciences Beijing 100049, China

E-mail: ylyan@iccas.ac.cn; yszhao@iccas.ac.cn

Table of Contents

1. Materials and experimental details.

1). Materials

2). Preparation of OPV-BODIPY pair doped PS microspheres

3). Characterizations

2. **Supplementary Figure 1.** Synthetic route of compound OPV.

3. **Supplementary Figure 2.** ^{1}H NMR of OPV molecules in $CDCl_3$.

4. **Supplementary Figure 3.** Emulsification process of OPV-BODIPY/PS/CH_2Cl_2 solution in water.

5. **Supplementary Figure 4.** SEM image of a typical dye doped PS microsphere.

6. **Supplementary Figure 5.** Electric field distribution of resonant cavity modes in a single microsphere.

7. **Supplementary Figure 6.** Confocal microscopy images collected at different focal planes of a typical microsphere.

8. **Supplementary Figure 7.** Schematic demonstration of experimental setup for optical characterization.

9. **Supplementary Figure 8.** Photostability of a representative FRET microlaser.

10. **Supplementary Figure 9.** Lasing performances of a typical OPV-doped microsphere.

11. **Supplementary Table 1.** Fitted lifetime components and corresponding relative weights for the OPV-doped microsphere under different pump fluences.

12. **Supplementary Figure 10.** PL decay profiles for OPV-doped microspheres and OPV-BODIPY pair doped microspheres.

13. **Supplementary Figure 11.** PL spectra of microlasers with different doping molar fraction of BODIPY to OPV (f_{BODIPY}) under different pump energies.

14. **Supplementary Figure 12.** Schematic diagram of the conversion of quaternary keys

into binary keys.

Experimental Procedures

Materials. 4,4-difluoro-8-cyano-1,2,3,5,6,7-hexamethyl-4-bora-3a,4a-diaza-s-indacene (BODIPY) were purchased from TCI (Shanghai, China). 1,4-bis(α-cyano-4-diphenylaminostyryl)-2,5-diphenylbenzene (OPV) used in this work were synthesized with Knoevenagel condensation reactions (Figure S1-2).

Preparation of OPV-BODIPY pair doped PS microspheres. OPV-BODIPY pair doped PS microspheres were prepared through an emulsion-solvent-evaporation method. In a typical preparation, 50 μL well-mixed OPV-BODIPY/PS/dichloromethane (CH_2Cl_2) solution was added into 500 μL CTAB aqueous solution (2 mmol), which was subsequently treated with vigorous stirring. After aging for 2h, OPV-BODIPY pair doped PS microspheres were obtained in the colloid solutions. Later, the surfactant CTAB was removed through filtration and washing. The precipitate was redispersed in aqueous solution and then used to prepare samples for further characterizations by drop-casting. The diameters of obtained spheres can be well tuned from 3 to 20 μm through increasing the concentration of PS from 10 to 50 mg mL^{-1}.

Characterizations. ^{1}H NMR spectrum of OPV in $CDCl_3$ was recorded on a Bruker AVIII 400 MHz NMR Spectrometer. MS spectrum of OPV was recorded on a Bruker AUTO FLEXIII. Absorption and fluorescence spectra were measured on a UV-visible spectrometer (Shimidazu UV-2600) and a fluorescence spectrometer (Hitachi F-7000), respectively. Confocal images of the microspheres were recorded with a laser confocal fluorescence microscope (Olympus FV1000-IX81) equipped with a 405 nm laser. Light amplification was investigated with a home-built far-field micro-PL system. The single microsphere was locally excited with a focused 400 nm femtosecond laser (fs-laser), which was generated from the second harmonic of the fundamental output of a regenerative amplifier (Solstice, Spectra-Physics, 800 nm, 100 fs, 1 kHz). The microspheres on glass substrates (refractive index ~1.5) were excited with the laser beams through an objective (Nikon CFLU Plan, 50×, N.A. = 0.8), with input power altered by neutral density filters. The excitation laser was filtered with a 420 nm long-pass emission filter. PL signals from the collection point were dispersed with a grating and recorded with a thermal-electrically cooled CCD (Princeton Instruments, ProEm: 1600B). Time-resolved photoluminescence of the microlasers was measured by a streak camera (Hamamatsu photonics, C10910).

Results and Discussion

Synthesis of model compounds

Materials:

2,5-Dibromobenzene-1,4-dicarbaldehyde was purchased from Innochem Science & Technology (Beijing, China). 1,4-Dibromo-2,5-dimethylbenzene, 4-(diphenylamino) benzaldehyde, potassium tertobutoxide, and tetra-butyl ammonium hydroxide were purchased from Sigma-Aldrich (Shanghai, China).

Synthesis of 1,4-bis(α-cyano-4-diphenylaminostyryl)-2,5-diphenylbenzene (OPV)

Step1: Synthesis of 2-(cyanomethyl)-4-(diphenylamino)benzene[1]

2-(Cyanomethyl)-4-(diphenylamino)benzene was prepared from 4-(diphenylamino) benzaldehyde upon treatment with tosylmethylisocyanide (TosMIC) and t-BuOK in a single step.

Step 2: Synthesis of OPV[2]

2-(Cyanomethyl)-4-(diphenylamino)benzene (0.21 mmol) and [1,1';4',1"] terphenyl-2',5'-dicarbaldehyde (0.1 mmol) were dissolved in tert-butanol (1.2 mL) and THF (0.8 mL) under a nitrogen atmosphere. Potassium tert-butoxide (0.02 mmol) and tetra-ntbutylammonium hydroxide (0.02 mmol, 1 M solution in methanol) were added quickly; then, the mixture was stirred vigorously at 50 °C. Twenty mins later, the mixture was poured into acidified methanol. The crude product was precipitated from methanol and further purified by column chromatography in darkness. ^{1}H NMR (400 MHz, 25 °C, $CDCl_3$, TMS, ppm): δ = 8.235 (s, 2H), 7.530-7.464 (m, 8H), 7.431-7.392 (m, 8H), 7.306-7.251 (t, 8H), 7.131-7.106 (d, 8H), 7.102-7.073 (t, 4H), 7.051-7.032 (d, 4H). These are in great agreement with previous results,[2] demonstrating the successful synthesis of OPV compound with high purity. MALDI-TOF MS: m/z) 819.21 ($[M+H]^+$); calcd for $C_{60}H_{42}N_4$, 818.34.

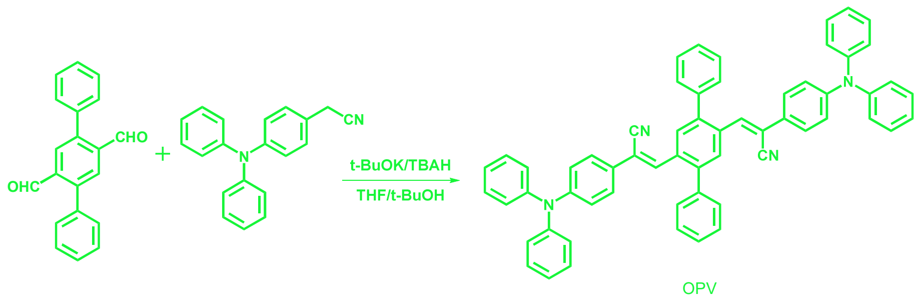

Supplementary Figure 1. Synthetic route of compound OPV.

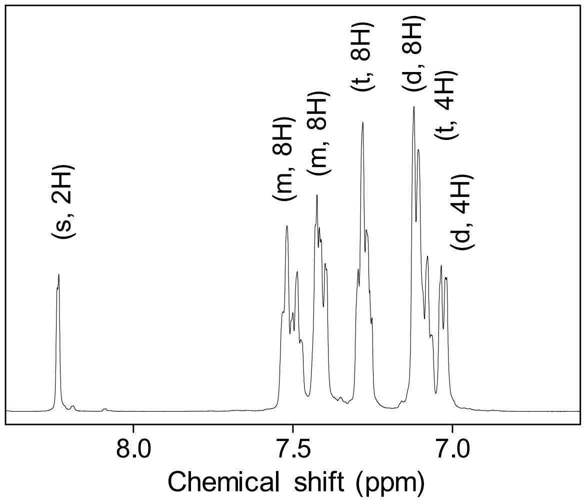

Supplementary Figure 2. ^1H NMR of OPV in $CDCl_3$. s, singlet; d, doublet; t, triplet; m, multiplet.

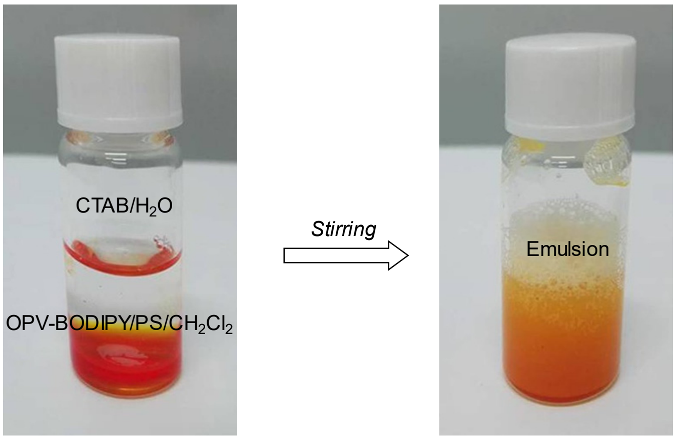

Supplementary Figure 3. Emulsification process of OPV-BODIPY/PS/CH_2Cl_2 solution in water.

With vigorous stirring, the clear interface between the CTAB aqueous solution and OPV-BODIPY/PS/CH_2Cl_2 solution completely disappeared and a stable oil-in-water emulsion was formed.

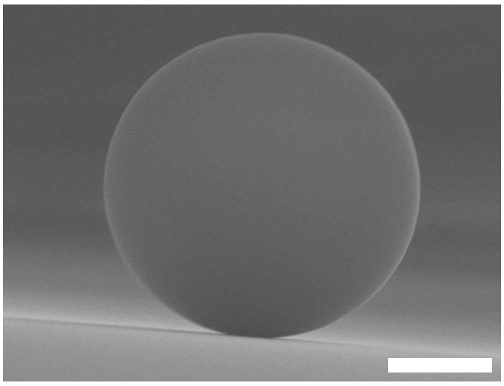

Supplementary Figure 4. SEM image of a typical dye doped PS microsphere. Scale bar is 2 μm.

As shown in Supplementary Figure 4, the acquired structure has perfect circle boundary and ultra-smooth surface, which are favorable for the whispering-gallery-mode (WGM) resonance,[3] possibly triggering a low-threshold WGM lasing emission.

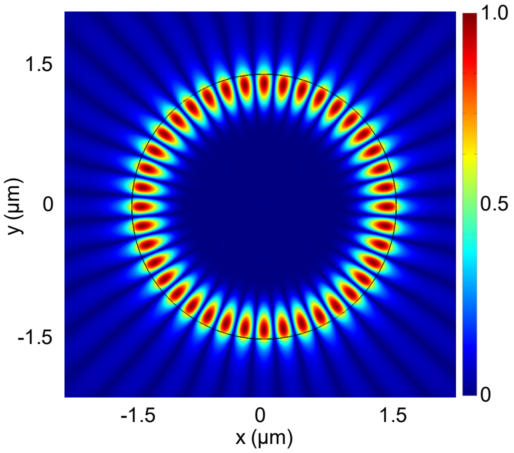

Supplementary Figure 5. Electric field distribution of resonant cavity modes in a single microsphere.

The electric field distribution reveals that the energy of the photons (λ = 630 nm, in the gain range of the BODIPY dye doped microsphere) can be well confined within the microsphere (group refractive index = 1.59), and the radial scattering into air is quite limited. The optical mode profiles along the edge of the microspheres clearly show the efficient light guiding, which indicates a typical WGM-type resonance.

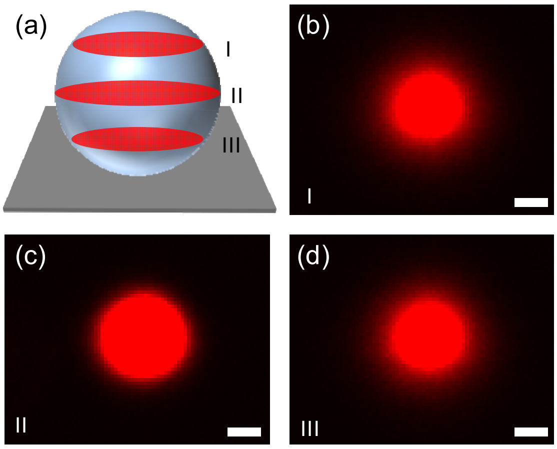

Supplementary Figure 6. Confocal microscopy images collected at different focal planes of a typical microsphere. (a) Schematic diagram for the confocal microscopy images (emission band, 620-650 nm) collected at different focal planes of the microsphere (interplane distance, 2 μm). (b-d) The confocal microscopy images corresponding to the I-III focal planes in Figure 6a. Scale bars are 2 μm.

Figure S6a depicts schematic diagram for the confocal microscopy images (emission band, 620-650 nm) collected at different focal planes of a typical microsphere (I-III, interplane distance, 2 μm). As displayed in Supplementary Figure 6b-d, the confocal microscopy images recorded at different focal planes of the microsphere exhibit uniform red emission from BODIPY, indicating that acceptor molecules are well dispersed within the microspheres.

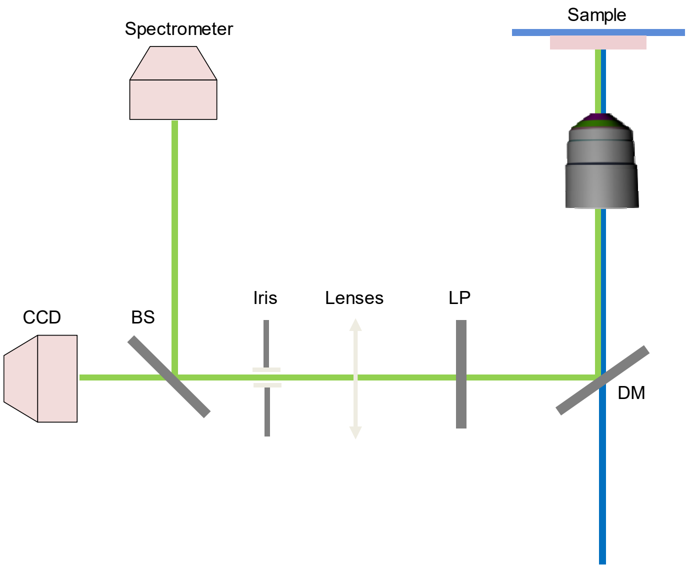

Supplementary Figure 7. Schematic demonstration of experimental setup for optical characterization.

A homemade micro-photoluminescence system was used to examine optical properties of the composite microspheres. These microspherical resonators were dispersed on a glass substrate (refractive index about 1.5). An objective lens (50×, N. A. = 0.8) was used to focus the pump beam (400 nm, 150 fs) down to a 2 μm-diameter spot and collect PL signals. After passing through a dichroic mirror (DM 400 nm) and an emission filter (420 nm LP), the collected PL signal was focused by a group of lenses onto a removable iris. The output signal was spatially selected by the iris and analyzed with a spectrometer.

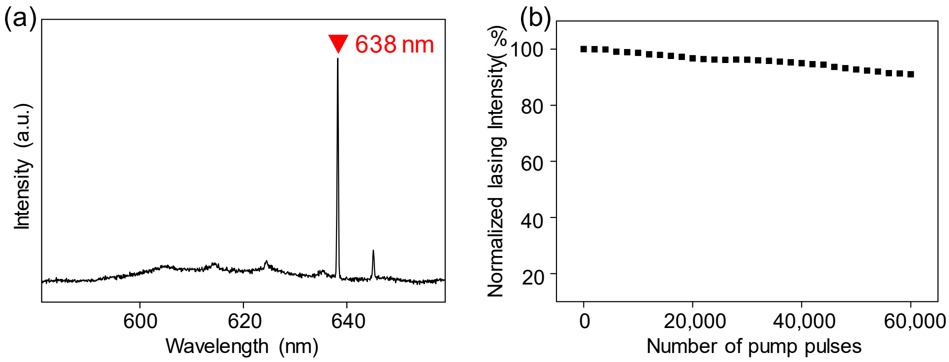

Supplementary Figure 8. Photostability of a representative FRET microlaser. (a) Lasing spectra of the FRET microlaser. (b) Normalized lasing emission intensity (at ~638 nm) as a function of pump pulse number for the FRET microlaser.

We selected a representative microsphere to evaluate the photosability of the FRET microlasers. When the dye-doped microsphere was excited locally at the pump fluence of 163 nJ cm^{-2} (above the lasing thresholds), the PL intensity in the gain region were dramatically amplified (Figure S8a), manifesting lasing action from the dye doped microstructure. We then recorded the lasing emission intensity at 638 nm in terms of number of excitation pulses. As displayed in Figure S8b, the lasing emission intensity decreased by less than 10% after 60,000 pump pulses, which demonstrates high photostability under laser operation. Such a high photostability may result from the robust microcavity and efficient FRET process in the microspheres.

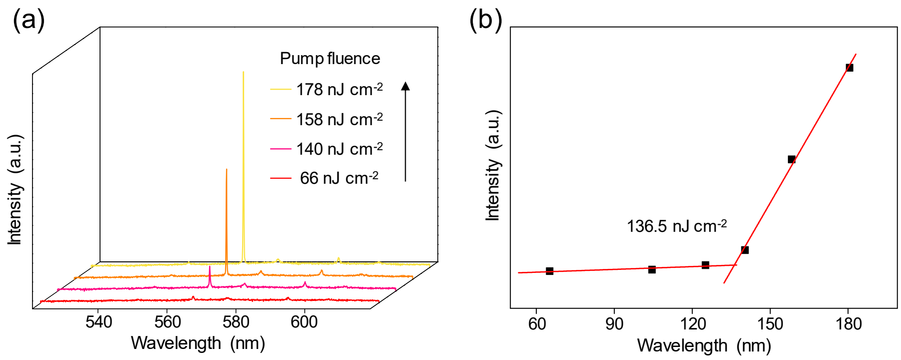

Supplementary Figure 9. Lasing performances of a typical OPV-doped microsphere. (a) PL spectra of an individual OPV dyes-doped microsphere under different pump energies. (b) Power-dependent profiles of PL intensities around the mode peak 565 nm.

With increasing pump fluence, the PL intensity in the gain region were dramatically amplified, manifesting lasing action from the OPV doped microstructure (Figure 9a). The peak emission intensity increased rapidly and superlinearly with the pump fluence, suggesting a clear knee behavior at 136.5 nJ cm^{-2} (Figure 9b). This nonlinear gain behavior clearly confirmed lasing action of the OPV-doped microspheres.

Supplementary Table S1. Fitted lifetime components and corresponding relative weights for the OPV-doped microsphere monitored at 565nm under different pump fluences.

	t_1 (ps)	w_1	t_2 (ps)	w_2	χ^2	t_{av} (ps)
0.10 P_{th}	1634	1.00	-	-	0.98	1624
1.12 P_{th}	1670	0.02	73	0.98	0.99	105
1.49 P_{th}	1568	0.01	57	0.99	0.97	72
2.01 P_{th}	1565	0.003	41	0.997	0.97	45

The PL decay curves were fitted to multiple exponential functin $I = A\ (w_1e^{-t/t_1} + w_2e^{-t/t_2} + \cdots) + B$, where I is the fluorescence intensity of OPV, A is the intensity constant, and B is the instrumental background, respectively. The average lifetime (t_{av}) was calculated according to the formula $t_{av} = w_1t_1 + w_2t_2 + \cdots$. When the pump power density is smaller than P_{th}, the PL emission follows a single-exponential decay with an average lifetime τ_D of 1.6 ns, corresponding to the spontaneous decay process. Once the excitation fluence increased above the lasing threshold, the PL signal turns to decay biexponentially. Besides the initial slow component (~1.6 ns), a new rapid component (<80 ps) emerges, which corresponds to the stimulated emission transition depleting excited-state population instantly.[4] The decay process accelerates as the pump power increases, leading to the average decay time decreased from about 1.62 ns to 45 ps.

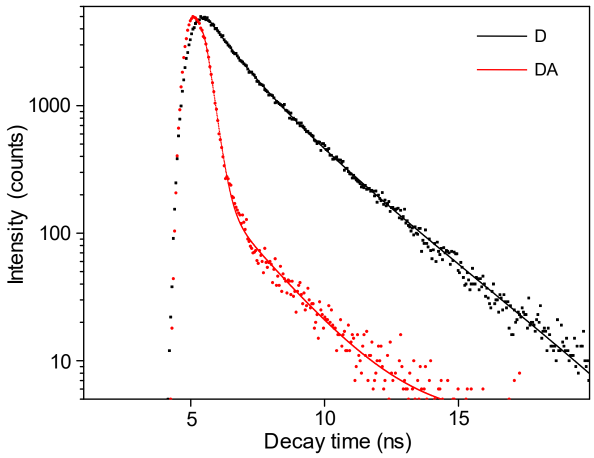

Supplementary Figure 10. PL decay profiles for OPV-doped microspheres and OPV-BODIPY doped microspheres.

As shown in Supplementary Figure 10, the PL decay in OPV-BODIPY doped microspheres becomes obviously faster than that in OPV doped microspheres. The average lifetime of OPV in the absence of BODIPY was determined to be 1.68 ns, while it decreased to 0.52 ns in the presence of BODIPY. The sharp shortening of the OPV lifetime in OPV-BODIPY doped microspheres validates that energy transfer from OPV to BODIPY occurred. The rate constant of FRET were calculated according to $k_{FRET} = 1/\tau_{DA} - 1/\tau_{D}$, where τ_{DA} is the lifetime of donor in presence of acceptor molecules, and τ_{D} is the lifetime of donor OPV in absence of acceptor BODIPY, respectively. Based on the PL decay profiles, the τ_{DA} and τ_{D} are 0.52 ns and 1.68 ns, respectively. Therefore, the rate constant of FRET was determined to be 1.3×10^{9} s^{-1}. Furthermore, the energy transfer efficiency ($\varnothing$) between OPV and BODIPY molecules was estimated to be 69.1% based on the equation $\varnothing = 1\text{-}\tau_{DA}/\tau_{D}$, where τ_{DA} and τ_{D} are the lifetime values in the presence and absence of BODIPY molecules.

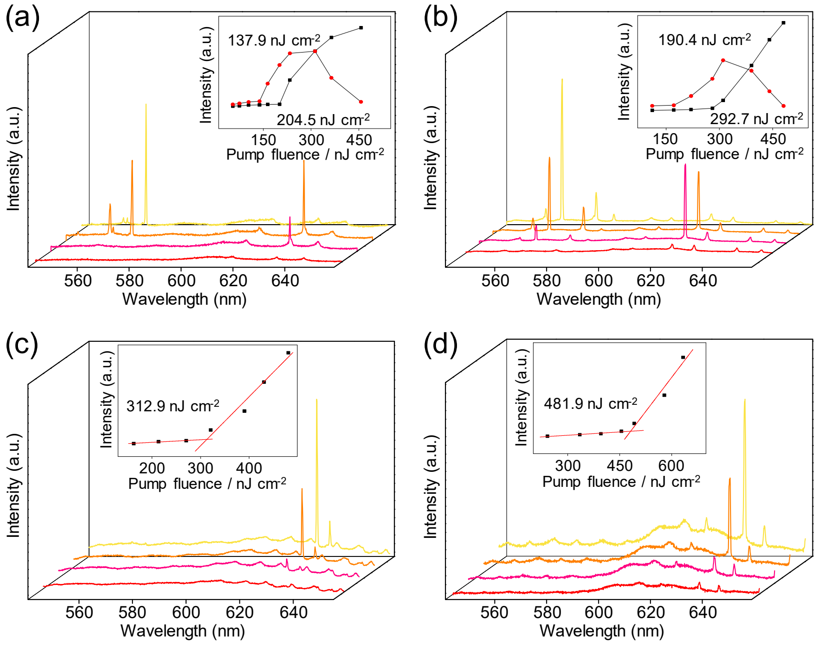

Supplementary Figure 11. PL spectra of microlasers with different doping molar fraction of BODIPY to OPV (f_{BODIPY}) under different pump energies. From a to d: f_{BODIPY} = 0.125 (a), 0.250 (b), 0.375 (c), and 0.500 (d), respectively. Insets show plots of PL intensity versus pump energies.

Considering K_{ET} is highly dependent on donor/acceptor proportion, we investigated K_{ET}-dependent lasing with microspheres of varied mole fractions (f_{BODIPY}, BODIPY relative to OPV). When the f_{BODIPY} increased from 0.125 to 0.250, lasing emission from the donor and acceptor can be obtained, implying K_{ET} is comparable with K_r in both cases. At the relative higher doping ratios such as 0.375 or 0.500, only the BODIPY lasing emission was observed as a result of the larger K_{ET} than K_r. Therefore, the microspheres with different doping ratios would exhibit diverse emission states under a fixed pump fluence, which should be ascribed to the distinct balance between individual K_{ET} and K_r, providing a good platform for optical encoding and cryptographic implementation.

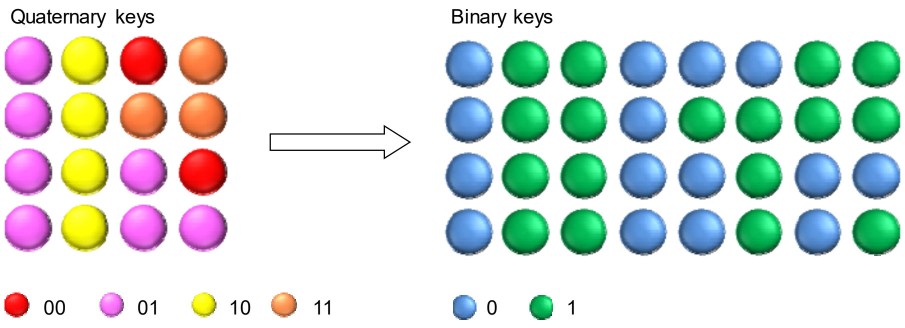

Supplementary Figure 12. Schematic diagram of the conversion of quaternary keys into binary keys.

The quaternary keys can be converted to binary keys with a larger key size to better use the embedded information. As shown in the Supplementary Figure 10, every quaternary bit can be converted to two binary bits. Therefore, the quaternary keys can significantly enhance the security level of the cryptographic keys without increasing the physical size of the bit array.

References

1. He F., Tian L. L. and Tian X. Y. *et al.* Diphenylamine-Substituted Cruciform Oligo(phenylene vinylene): Enhanced One- and Two-Photon Excited Fluorescence in the Solid State. *Adv. Funct. Mater.* 2007, **17**, 1551-7.

2. Li Y., Shen F. and Wang H. *et al.* Supramolecular Network Conducting the Formation of Uniaxially Oriented Molecular Crystal of Cyano Substituted Oligo(p-phenylene vinylene) and Its Amplified Spontaneous Emission (ASE) Behavior. *Chem. Mater.* 2008**, 20**, 7312-8.

3. Ta V. D., Chen R. and Sun H. D. Self-Assembled Flexible Microlasers. *Adv. Mater.* 2012, **24**, OP60-4.

4. Eaton S. W., Lai M. and Gibson N. A. *et al.* Lasing in Robust Cesium Lead Halide Perovskite Nanowires. *Proc. Natl. Acad. Sci. U. S. A.* 2016, **113**, 1993-8.
